# Transmission of *Mycobacterium tuberculosis* between Farmers and Cattle in Central Ethiopia

**DOI:** 10.1371/journal.pone.0076891

**Published:** 2013-10-10

**Authors:** Gobena Ameni, Konjit Tadesse, Elena Hailu, Yohannes Deresse, Girmay Medhin, Abraham Aseffa, Glyn Hewinson, Martin Vordermeier, Stefan Berg

**Affiliations:** 1 Animal Health and Zoonotic Research Unit, Aklilu Lemma Institute of Pathobiology, Addis Ababa University, Addis Ababa, Ethiopia; 2 TB Research Team, Armauer Hansen Research Institute, Addis Ababa, Ethiopia; 3 TB Research Group, Animal Health and Veterinary Laboratories Agency, New Haw, Addlestone, Surrey, United Kingdom; Auburn University, United States of America

## Abstract

**Background:**

Transmission of *Mycobacterium tuberculosis* (*M. tuberculosis*) complex could be possible between farmers and their cattle in Ethiopia.

**Methodology/Principal Findings:**

A study was conducted in mixed type multi-purposes cattle raising region of Ethiopia on 287 households (146 households with case of pulmonary tuberculosis (TB) and 141 free of TB) and 287 herds consisting of 2,033 cattle belonging to these households to evaluate transmission of TB between cattle and farmers. Interview, bacteriological examinations and molecular typing were used for human subjects while comparative intradermal tuberculin (CIDT) test, post mortem and bacteriological examinations, and molecular typing were used for animal studies. Herd prevalence of CIDT reactors was 9.4% and was higher (p<0.01) in herds owned by households with TB than in herds owned by TB free households. Animal prevalence was 1.8% and also higher (p<0.01) in cattle owned by households with TB case than in those owned by TB free households. All mycobacteria (141) isolated from farmers were *M. tuberculosis*, while only five of the 16 isolates from cattle were members of the *M. tuberculosis* complex (MTC) while the remaining 11 were members of non-tuberculosis mycobacteria (NTM). Further speciation of the five MTC isolates showed that three of the isolates were *M. bovis* (strain SB1176), while the remaining two were *M. tuberculosis* strains (SIT149 and SIT53). Pathology scoring method described by “Vordermeier *et al.* (2002)” was applied and the average severity of pathology in two cattle infected with *M. bovis*, in 11 infected with NTM and two infected with *M. tuberculosis* were 5.5, 2.1 and 0.5, respectively.

**Conclusions/Significance:**

The results showed that transmission of TB from farmers to cattle by the airborne route sensitizes the cows but rarely leads to TB. Similarly, low transmission of *M. bovis* between farmers and their cattle was found, suggesting requirement of ingestion of contaminated milk from cows with tuberculous mastitis.

## Introduction

*M. tuberculosis* and *M. bovis* are amongst the most important pathogens from the MTC, a highly related group of mycobacteria that cause TB in humans and other mammals [2]. *M. tuberculosis* is mainly considered as a human pathogen causing active TB in approximately eight million people every year [3], whereas *M. bovis* has a broader host range responsible for TB in domestic and wild animals [4]. It is well established that *M. bovis* also infects humans, causing zoonotic TB in humans [5].

According to the World Health Organization (WHO) Ethiopia ranks seventh in the world among the 22 high-burden TB countries, with an estimated incidence rate of 378 cases per 100,000 population of which ∼1/3 are reported as new smear positive cases [6]. Smear positive cases are infectious and could be the sources of infection for healthy humans and animals. Although a recent comprehensive study on the proportion of human TB caused by *M. bovis* in Ethiopia suggested a rate less than 1% [7], smaller studies have reported higher isolation rates of *M. bovis* from humans [8]. The main routes of *M. bovis* transmission from infected animal to humans are believed to be through ingestion of raw milk and/or inhalation of aerosol from diseased animal, mainly in settings where pasteurization of milk is not widely established. Different studies have reported isolation of *M. tuberculosis* from domestic and wildlife animals [9]–[19]. The source of *M. tuberculosis* in animals is most frequently considered to be active TB patients expelling *M. tuberculosis* through sputum primarily, less often through urine or feces [11]–[14].

In developing countries, the dietary habit of people, close physical contact between humans and animals, rise in the incidence of immunosuppressive diseases, and inadequate disease control measures in animals and humans facilitate the transmission of the disease between animals and humans [5]. The present study took place in central Ethiopia where the majority of inhabitants in this area engaged in agriculture. As the climate condition is suitable for cattle production, these farmers practice mixed cattle farming in addition to crop cultivation. Previous studies conducted on bovine TB in herds in the study area have indicated high prevalence [20]–[22] of the disease. Thus, MTC prevalence in both human and cattle in the area suggest the possibility of existence of transmission between farmers and their cattle. The transmission of TB between farmers and their cattle could be associated with habits of farmers such as use of chewing tobacco for worming which is widely practiced in the area [19]. Therefore, this study was conducted to investigate the transmission of MTC between cattle and their owners in the area. To achieve this study, farmers with active TB were recruited from health institutions. TB free farmers living in the same village were identified to serve as control. This was followed by tracing and investigating cattle herds owned both groups of farmers.

## Materials and Methods

### Study Design

We hypothesize that the transmission of MTC between farmers and their cattle is prevalent in central Ethiopia. To prove or disprove this hypothesis, a case-control study (human TB cases and TB free controls) with two human and two linked animal cohort was conducted. The human risk factor study was a retrospective cohort study while the cattle study aspect was a prospective cohort study. The cases were farmers who visited health institutions in the area and were diagnosed as smear positive pulmonary TB patients by the health personnel. Households in which TB cases live were considered to be TB positive households. The controls were farmers residing in the households located in same village with TB positive households but did not have history of TB for the last decade. Households in which TB cases did not occur for the last decade were considered as TB negative households. Identification of the cases was done at health institutions while identification of controls was made in the villages after tracing the resident cases. Cases were defined on the basis of clinical and laboratory examination while controls were defined on the basis of interview and clinical examination. A total of 146 cases and 141 controls were recruited in the study. After identification of cases and controls, the herds were tested by CIDT for bovine TB. Thus, 146 “case” herds and 141 “control” herds were tested.

### Study Subjects

**Figure 1:**
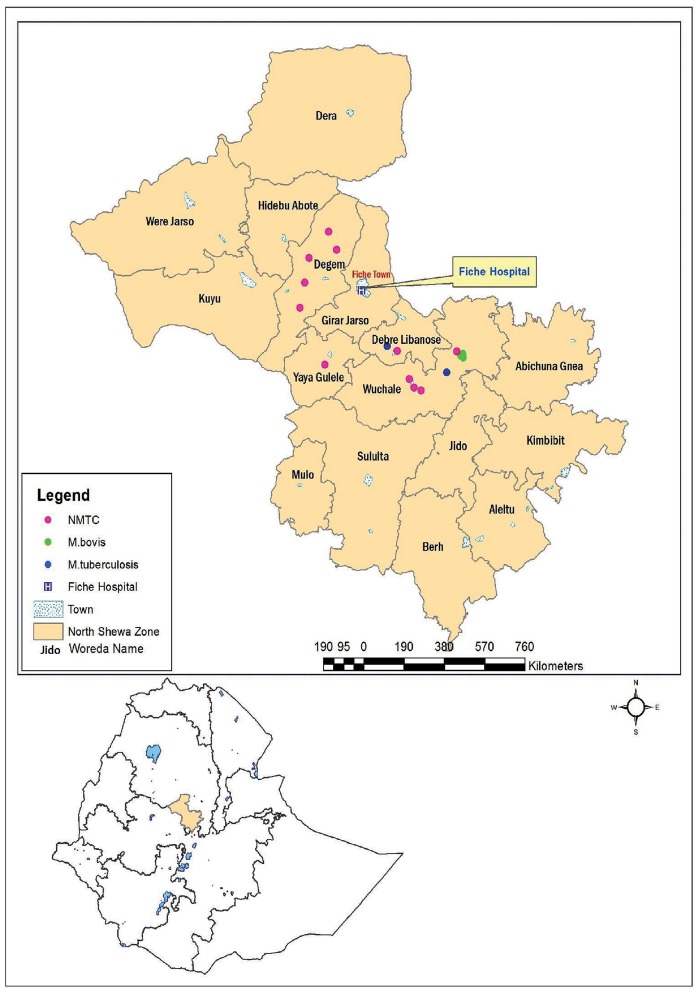
Distribution and frequencies of mycobacteria isolated from tissues of skin test positive cattle in central Ethiopia. Majority (68.8%) of the isolates were NTMs (pink dots on the map) while 18.8% were *M. bovis* (green dots) and 12.6% were *M. tuberculosis* (blue dots).

The study was conducted in central Ethiopia (Figure 1). Fiche Hospital and district health centers were used for the identification of households with TB cases. Farmers with active TB were identified in the health institutions, consented and requested for submission of sputum samples before treatment. The sputa samples were collected and examined for acid fast bacilli (AFB) as routine diagnostic procedure. Leftover sputa of the AFB positive farmers were transported to TB laboratory of the Aklilu Lemma Institute of Pathobiology for mycobacterial culturing. This was followed by tracing the herds of households from where AFB positive farmers come and testing using CIDT. Side by side, herds belonging to households free from TB were identified and tested with CIDT for bovine TB. Using this procedure, 287 households (146 households with pulmonary TB and 141 TB-free households) and 2033 cattle owned by both groups were investigated. Human TB positivity was defined by routine diagnostic procedures including clinical examination and acid-fast staining. History and clinical examination were used for screening of TB free farmers residing in the same village with farmers with pulmonary TB. Both TB positive and TB negative households keep cattle for multi-purpose. Female cattle were kept for reproduction and milk production while male cattle were kept for crop production. Both female and male cattle were kept mixed together and as such there was no specialization of cattle production either as dairy or as beef cattle. CIDT was performed on both female and male cattle. During testing parameters such as age, sex, body condition score were recorded for each of the study cattle.

### Interview of Cattle Owners

A total of 287 individuals from the same number of households (usually heads of the households) were interviewed. Of these 287 individuals, 146 were TB positive while the remaining 141 were TB negative. These individuals were interviewed by their local language. The interview consisted of closed and open questions which address the knowledge, attitude and practices for the farmers in relation to TB in humans and cattle (see attached annex).

### Culturing of Sputum

A total of 146 sputum samples were collected in sterile universal tubes from smear positive pulmonary TB patients using sterile leak proof disposable plastic material. The sputum samples were processed (decontaminated and neutralized) for mycobacterial culturing according the standard operating procedure described earlier by WHO [23]. Thereafter, 100 µl neutralized sample was inoculated onto two slants of Lowenstein-Jensen (LJ) media, one supplemented with pyruvate and one with glycerol, and incubated at 37°C for up to 5–8 weeks. Growth of mycobacteria was monitored every week for up to 8 weeks. Sample negative for AFB after 8 weeks of growth were considered negative.

### Comparative Intradermal Tuberculin (CIDT) Testing of Cattle

A total of 287 herds consisting of 2033 cattle (1063 owned by TB positive households and 970 owned by TB negative households) were tested by CIDT test [24]. Two sites, separated by 12 cm, on the middle the left neck were shaved and skin thickness was measure with a caliper. Aliquots of 0.1 ml of 2500 IU per ml of avian purified protein derivative (PPD) and 0.1 ml of 2000 IU per ml of bovine PPD (Lelystad, The Netherlands; Animal Health and Veterinary Laboratories Agency, Weighbridge, UK) were injected to the dermis of the study animals. The skin thickness at each injection site was measured again after 72 hours. The interpretation of the result was made on the basis of the difference in skin thickness at the bovine and avian PPD injection sites. Two cut-off values were used to determine BTB status of an animal; the animal was considered as positive for BTB if the skin thickness at bovine PPD injection site minus the skin thickness at avian PPD injection site was >4 mm [23] or if the difference was >2 mm [20].

### Pathological Examination

Of the total 2,033 cattle subjected to CIDT test, 36 were found to be strong reactors and thus slaughtered for investigation. The lungs and lymph nodes of these 36 CIDT positive cattle were removed and examined for the presence of gross TB lesions. Each of the seven lobes of the lungs were thoroughly inspected and palpated for any suspicious gross TB lesions. Similarly, mandibular, retropharyngeal, cranial and caudal mediastinal, left and right bronchial, hepatic, and mesenteric lymph nodes were sliced into thin sections and inspected for the presence of visible lesions according to the protocol described earlier [1], [25]. When gross lesions suggestive of BTB were found in any of the tissues, the animal was classified as having lesions. Tissues with visible lesions were collected and processed for bacterial isolation using culture. Tissues with non-visible lesions were not cultured.

### Culturing of Suspicious Tissue Lesion

A total of 33 tissues with suspicious lesions were collected from 36 necropsied cattle into universal bottles containing 5 ml of sterile 0.9% saline and transported in cold chain into the laboboratories for culturing. Each tissue was divided into two and processed for culturing in two laboratories, namely the Aklilu Lemma Institute of Pathobiology (ALIPB) and the Armauer Hansen Research Institute. In the laboratories, the specimens were sectioned using sterile blades and then homogenized with a mortar and pestle. The homogenate samples were processed (decontaminated and neutralized) for mycobacterial culturing according the OIE standard operating procedure [24]. Thereafter, 100 µl neutralized sample was inoculated onto two slants of Lowenstein-Jensen (LJ) media, one supplemented with pyruvate and one with glycerol, and incubated at 37°C for up to 5–12 weeks. Growth of mycobacteria was monitored every week for up to 12 weeks. Sample negative for AFB after 12 weeks of growth were classified as negative.

### Molecular Typing of Mycobacteria

A total of 24 acid-fast bacilli (AFB) positive cattle isolates were heat-killed by mixing 2 loop-full of colonies in 400 µl distilled H_2_O followed by incubation at 80°C for 1 h. Thereafter, they were subjected Genus typing using multiplex polymerase chain reaction (PCR) to detect the presence of the Genus *Mycobacterium* in the isolate and to differentiate *M. tuberculosis* complex from the other mycobacterial species [26]. Region of difference-based RD4 and RD9 deletion typing were used for identification of *M. tuberculosis* and *M. bovis* according to previous protocol [18]. Spoligotyping was performed on 139 human and five cattle isolates following the procedure as described previously [27].

### Ethical Consideration

Both the human and animal components of the project were approved by the Ethical Clearance committee of the Aklilu Lemma Institute of Pathobiology (ALIPB/IRB/03/2009–10). All owners gave written consent to participate in the study and permission for their cattle to be included. All cattle that were recruited in the study were de-wormed after the PPD testing on free basis. TB positive cattle were slaughtered humanely in the Fitche Abattoir following the routine ante mortem and post mortem procedures.

### Data Analysis

Cattle owners were classified in two categories; as households identified as TB positive (case group) and those that were TB negative (control group). The two categories were compared in terms of selected household and animal characteristics using chi-square. Crude and adjusted effects of TB positivity of the owner on the tuberculin test result of cattle were investigated using logistic regression. The clustering effect that could result from the fact that many animals being owned by the same owner was considered in logistic regression modeling.

## Results

### Herd and Animal Prevalence

A total of 2,033 cattle, 1063 owned by case households with TB and 970 owned by control households not diagnosed with TB, were CIDT tested in 287 herds from five districts. Two cut-off values of skin test result (>4 mm and >2 mm) were employed for the estimation of the prevalence at household/herd and animal levels. Table 1 shows the herd prevalence of BTB using these two cut-off values in herds owned by households with TB patients and in herds owned by TB free households from the same village. The overall herd prevalence was 9.4% at cut-off >4 mm and 20.8% at cut-off >2 mm. At both cut-off values the CIDT-positive herd prevalence was significantly greater in herds owned by households with TB patients than in herds owned by TB free households living in the same village. The overall animal prevalence was 1.8% (36/2033) at a cut-off >4 mm while it was 4.7% (96/2033**)** at a cut-off >2 mm. As for the herd prevalence, animal prevalence was significantly greater at both cut-off points in cattle owned by households with TB patients than in cattle owned by TB free individuals households from the same village (Table 1).

**Table 1:** Bovine tuberculosis prevalence in herds and animals owned by households with TB patients and TB free households in the same village.

	>4 mm cut off			>2 mm cut-off		
Household status	TB positive household	TB negative household	Total	TB positive household	TB negative household	Total
**Herd prevalence**						
Number of herds examined	146	141	287	146	141	287
Number of herds positive	21	6	27	39	19	58
Prevalence (95%CI)	14.4% (9.1,21.1)	4.3% (1.9, 9.0)	9.4% (6.3, 13.4)	26.7% (19.7, 34.7)	13.5% (8.3, 20.2)	20.2% (15.7, 25.3)
**p-value**	**<0.01**			**<0.01**		
**Animal prevalence**						
Number cattle examined	1,063	970	2,033	1,063	970	2,033
Number cattle positive	30	6	36	75	21	96
Prevalence (95%CI)	2.8% (1.9, 4.0)	0.6% (0.2, 1.3)	1.8% (1.2, 2.4)	7.1% (5.6,8.8)	2.2% (1.3,3.3)	4.7% (3.8, 5.7)
**p-value**	**<0.001**			**<0.001**		

### Risk Factors in Cattle Owners

A comparative assessment of different risk factors was made between TB positive (case) and TB negative (control) farmers and the results of selected characteristics of study participants are summarized in Table 2. Cattle owner who was TB positive was more likely to share house with the animals, more likely to have PPD positive animals, and more likely to have awareness about TB than the TB free control group living in the same village.

**Table 2:** Comparison of risk factors of tuberculosis between farmers with pulmonary tuberculosis and tuberculosis free farmers in central Ethiopia.

Characteristics of respondent	Number (%)		?^2^	p-value
	TB cases (n = 146)	Controls (n = 141)		
Sex			35.3	<0.001
Male	74(50.7)	118(83.7)		
Female	72(49.3)	23(16.3)		
Age [mean(sd)]	34.7(1.3)	44.0(1.3)	5.07	<0.001
Consumption of raw milk	121(82.9)	115(82.1)	0.03	>0.05
Taking medication is a means to cure from TB	4(4.1)	27(19.3)	16.12	<0.001
Tobacco chewing			1.14	>0.05
Yes	25(17.1)	31(22.1)		
No	21(82.9)	109(77.9)		
Tobacco could transmit TB from human to animal	60(41.1)	59(42.1)	0.03	>0.05
Share house with animals			7.93	<0.01
Yes	94(64.4)	67(47.9)		
No	52(35.6)	73(52.1)		
Owns zebu breed	130(89.0)	123(87.2)	0.22	>0.05
Owns cross breed	96(65.8)	106(75.7)	3.42	>0.05
Owns Holstein breed	7(4.8)	10(7.2)	0.73	>0.05
Animals grazing on field			0.32	>0.05
Yes	137(93.8)	129(92.1)		
No	9(6.2)	11(7.9)		
Some of his/her animals are positive for TB	21(14.4)	6(4.3)	8.63	<0.01
Thinks that he/she know TB	90(61.6)	48(34.3)	21.4	<0.001
Has TB positive family member	58(39.7)	10(7.1)	41.9	<0.001
Know symptoms of TB	115(78.8)	63(45.0)	34.7	<0.001
Know cattle can be infected with TB	29(19.9)	59(42.1)	16.7	<0.001
Thinks that TB can be transmitted from cattle to human	40(27.4)	49(35.0)	1.93	>0.05
Thinks that animals can acquire TB from humans	49(33.6)	36(25.7)	2.12	>0.05

The result of logistic regression taking skin test as a binary outcome and TB status of the owner (confirmed positive versus negative as main exposure variable) is summarized in Table 3. The risk for an animal being positive for TB as measured by skin test result was significantly higher (crude OR = 4.52; 95%CI, 1.80–11.36 at >4 mm cut-off and crude OR = 3.32; 95%CI, 1.88–5.87 at 2 mm cut-off) in cattle owned by confirmed TB positive owner than in cattle owned by TB negative owner. These associations were not confounded by pre-specified characteristics of the owner (age, sex, tobacco treatment of cattle, sharing shelter with animals) and pre-specified characteristics of animals (sex, age, breed, body condition and field grazing practice) (Table 3).

**Table 3:** The effect of being owned by households with confirmed TB positive patients on skin test result of cattle in central Ethiopia.

Character	Cut-off >4 mm	Cut-off >2 mm
	OR(95%CI)	OR (95%CI)
Unadjusted effect of being owned by confirmed case compared to being owned by control	4.52(1.80, 11.36)	3.32(1.88, 5.87)
Effect of being owned by confirmed case compared to being owned by control adjustedfor owner characteristics:		
Sex of respondent	4.89(1.79, 13.36)	3.34(1.78, 6.29)
Age	4.57(1.71, 12.21)	3.50(1.86, 6.58)
Tobacco chewing	4.47(1.78, 11.21)	3.25(1.86, 5.70)
If they share houseWith animals	4.09(1.66, 10.09)	3.15(1.76, 5.63)
Knowledge levelAbout TB	4.76(0.51, 44.28)	3.46(0.89, 13.39)
Effect of being owned by confirmed case compared to being owned by control adjustedfor cattle characteristics:		
Sex	4.52(1.80, 11.31)	3.33(1.89, 5.89)
Age	4.44(1.79, 11.01)	3.37(1.92, 5.92)
Breed	4.62(1.82, 11.72)	3.35(1.89, 5.92)
Body condition	4.45(1.77, 11.19)	3.37(1.93, 5.90)
Grazing on field	4.79(1.94, 11.84)	3.40(1.95, 5.94)

After adjusting for TB status of the cattle owner (TB case or control), the risk of TB positivity (i.e. skin test result) was not significantly associated with pre-specified characteristics of the owner (age, sex, tobacco treatment of cattle, sharing shelter with animals, level of knowledge about TB) and animals (sex, age, bread, body condition and field grazing practice) (data not shown).

### Post Mortem and Bacteriological Findings

General data on each of the 36 slaughtered cattle are presented in Table 4. The selection of the slaughtered cattle was on the basis of reaction to tuberculin, mainly reaction to bovine PPD. The proportion of reactors with visible lesion was 69% (25/36). Culture positivity was recorded for 16 (44%) of the 36 reactors. The average severity of pathology in two cattle infected with *M. bovis* was 5.5 while the average severity of pathology in two cattle infected with *M. tuberculosis* was 0.5 (Table 4). On the other hand, the average severity of pathology in 14 cattle infected with NTM was 2.1. Results of the skin test, post mortem, culture and molecular typing are presented. Most of the isolates (11/16) were NTM. Only five of the isolates were members of *M. tuberculosis* complex.

**Table 4:** Overall characteristics of reactor cattle slaughtered for pathological and bacterial studies.

ID	District	Sex	Age (years)	Breed	Status of owner	BCS	*PPDA*	*PPDB*	*PPD* *(B–A)*	*Pathology*	*Tissues with lesion*	Culture (AFB+)	Species
C97c7	G.Jarso	M	10	Zebu	TB free	Lean	Reactor	Reactor	*5*	*2*	MS	Negative	Negative
P16c2	Wuchale	M	3	Cross	TB case	Medium	None	Reactor	6	9	MS, LB,RPH & RCL	Positive	2*M. bovis*
P16C4	Wuchale	M	2	Cross	TB case	Lean	None	Reactor	5	2	CRMD	Positive	*M. bovis*
P4C1	Wuchale	M	0.6	Cross	TB case	Lean	Reactor	Reactor	6	5	HP, MS	Positive	NTM
P4C6	Wuchale	F	7	Cross	TB case	Lean	Reactor	Reactor	3	1	RPH	Positive	NTM
P4C7	Wuchale	F	3	Cross	TB case	Lean	Reactor	Reactor	4	0	NVL	No result	Negative
629C3	Degem	M	6	Zebu	TB case	Lean	Reactor	Reactor	2	4	CAMD, MS	Positive	NTM
409C2	Wuchale	M	1.2	Zebu	TB case	Lean	None	Reactor	7	1	LDL	Positive	*M. tuberculosis*
409C4	Wuchale	F	1.5	Zebu	TB case	Lean	Reactor	Reactor	3	1	LB	Positive	NTM
409C10	Wuchale	M	5	Zebu	TB case	Medium	Reactor	Reactor	1	2	MS	Positive	NTM
520C1	Y. Gulele	M	4	Zebu	TB case	Lean	None	Reactor	6	0	NVL	No result	No result
1180C8	Y. Gulele	M	1	Zebu	TB case	Lean	Reactor	Reactor	6	0	NVL	No result	No result
1180C9	Y. Gulele	M	1	Zebu	TB case	Lean	Reactor	Reactor	4	1	CAMD	Positive	*M. tuberculosis*
723C1	Wuchale	M	4	Zebu	TB case	Lean	Reactor	Reactor	2	3	RCL	Negative	Negative
778C1	Degem	M	11	Cross	TB case	Lean	Reactor	Reactor	4	2	LB	Positive	NTM
1124C5	Y. Gulele	F	3	Zebu	TB case	Medium	Reactor	Reactor	7	3	MS	Negative	Negative
1124C6	Y. Gulele	F	7	Zebu	TB case	Medium	Reactor	Reactor	3	2	LDL	Positive	Negative
P44C5	Y. Gulele	M	8	Zebu	TB case	Medium	None	Reactor	4	2	MS	Positive	NTM
1300C 3	Y. Gulele	M	8	Zebu	TB case	Lean	None	Reactor	4	0	NVL	No result	No result
C13C1	Wuchale	M	7	Zebu	TB free	Medium	None	Reactor	5	3	MS	Negative	Negative
C16C7	Degem	M	5	Zebu	TB free	Lean	Reactor	Reactor	3	1	MS	Positive	NTM
CP25C5	Degem	M	12	Zebu	TB free	Lean	None	Reactor	7	2	HP, MS	Positive	NTM
CP301	Degem	M	5	Zebu	TB free	Medium	None	Reactor	5	4	CAMD, MS	Positive	NTM
Tag N06	D.libanos	F	7.8	Cross	TB case	Medium	None	Reactor	11	1	CAMD	Positive	NTM
Tag N14	D.libanos	F	4	Cross	TB case	Medium	Reactor	Reactor	5	0	NVL	No result	No result
Tag N03	D.libanos	F	1.5	Cross	TB case	Medium	Reactor	Reactor	5	0	NVL	No result	No result
Tag N04	D.libanos	F	1.7	Cross	TB case	Lean	Reactor	Reactor	3	0	NVL	No result	No result
Tag N07	D.libanos	F	2.5	Cross	TB case	Lean	None	Reactor	4	0	NVL	No result	No result
Tag N15	D.libanos	F	6	Cross	TB case	Medium	None	Reactor	6	2	MS	Negative	Negative
Tag N12	D.libanos	F	5	Cross	TB case	Lean	None	Reactor	3	0	NVL	No result	No result
35C5	Wuchale	F	6	Zebu	TB case	Lean	Reactor	Reactor	3	6	LCL, RDL, MS	Negative	Negative
P13C1	Wuchale	M	5	Zebu	TB case	Lean	Reactor	Reactor	4	0	NVL	No result	No result
P13C4	Wuchale	M	0.7	Cross	TB case	Lean	Reactor	Reactor	4	0	NVL	No result	No result
P13C6	Wuchale	F	9	Cross	TB case	Lean	Reactor	Reactor	4	3	MS	Negative	Negative
395C5	G.Jarso	F	6	Cross	TB case	Lean	Reactor	Reactor	1	1	MS	Negative	Negative
395C12	G.Jarso	M	10	Cross	TB case	Medium	Reactor	Reactor	3	3	CAMD	Negative	Negative

G. Jarso =  Girar Jarso, D. libanos =  Debre Libanos, Y. Gulele =  Yaya Gulele, MS =  Mesenteric lymph node (LN), LB =  left bronchial LN, RPH =  retropharyngeal LN, RCL =  right cardiac lobe, CRMD =  cranial mediastinal LN, HP = hepatic LN, CAMD =  caudal mediastinal LN, LDL =  left diaphragmatic lobe, LCL =  left cardiac lobe, RDL =  right diaphragmatic lobe, NTM, none-*M. tuberculosis* complex, BCS =  body condition, NVL, = Non-visible lesion.

### Molecular Characterization of Cattle Isolates

For identification of mycobacteria from farmers and their cattle molecular typing was used. Of the 24 cattle isolates (obtained from tissues of 16 cattle), 16 were positive for the Genus Mycobacterium. Out of these 16 isolates, five were members of the *M. tuberculosis* complex (MTC) while the remaining 11 were NTMs. Figure 1 shows the distribution of the different isolates in the study area. The five members of the MTC complex were further characterized and classified into three *M. bovis* isolates and two *M. tuberculosis* isolates.

**Figure 2:**
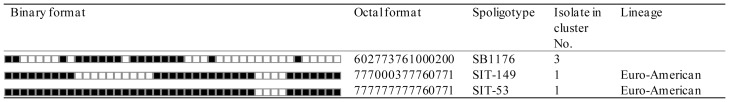
Spoligotype patterns of *M. tuberculosis* complex species isolated from cattle owned by farmers with active pulmonary tuberculosis in central Ethiopia. Three isolates of *M. bovis* were isolated from two oxen of a farmer with active pulmonary tuberculosis. These three isolates had the same spoligotype pattern and were SB1176. The other two isolates were *M. tuberculosis* and were from cattle owned by farmers with active tuberculosis. These isolates had different spoligotype pattern and were SIT149 and SIT53.

The *M. tuberculosis* and *M. bovis* isolates from cattle tissues were further characterized at strain level using spoligotyping (Figure 2). The spoligotype patterns of the two *M. tuberculosis* isolates were SIT149 and SIT53 (SpolDB4 database). On the other hand, the three *M. bovis* isolates exhibited the same pattern of *M. bovis* and they were clustered as a single type SB1176, which is a very dominant strain in central Ethiopia. The two cattle isolates of *M. tuberculosis* were members of the Euro-American lineage.

### Isolation and Molecular Characterization of *M. tuberculosis* Complex Isolates from Farmers

Culture positivity was observed in 97% (141/146) of the farmers with active pulmonary TB and confirmed AFB positive with Ziehl Neelsen staining. Of the 141 culture positive samples, 139 were confirmed to be *M. tuberculosis* isolates using RD9 deletion typing while the remaining two isolates did not give signal. These 139 isolates were characterized to the strain level by spoligotyping. Up on spoligotyping, 130 isolates gave good and interpretable patterns while the patterns of the remaining 9 isolates were poor and could not be interpreted.

**Figure 3:**
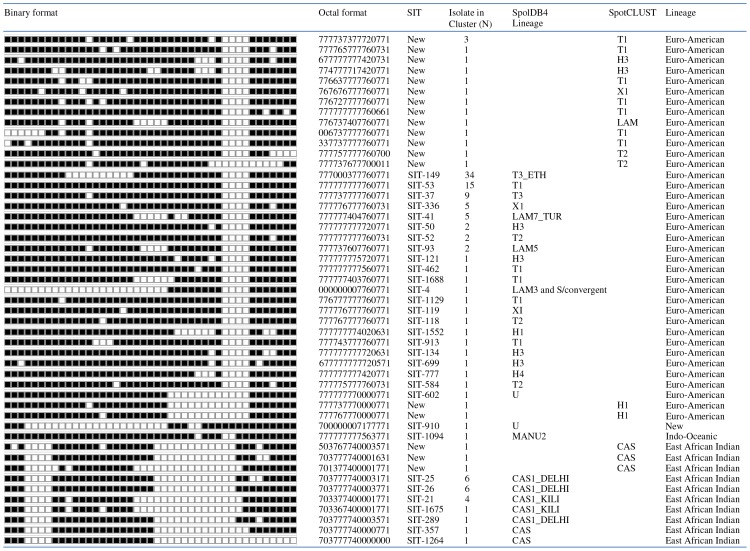
Spoligotype patterns of 130 *M. tuberculosis* isolated from pulmonary tuberculosis cases of farmers in central Ethiopia. The filled boxes represent the presence of spacers, and the empty boxes represent the absence of spacers. Spoligotyping of the 130 isolates produced 49 distinct patterns: Of these, 37 of had a unique pattern while the remaining 93 (71.5%) isolates were grouped into 12 clusters of strains possessing at least two isolates. The cluster size varied from 2 to 34 patients. Eighteen patterns of the 49 unique patterns were not previously reported. The most commonly patterns were SIT149, SIT53, and SIT37 each consisting of 34, 15, and 9 isolates, respectively. The most predominant lineage was Euro-American consisting of 78.5% of the isolates while 17.7% of the isolates were East African Indian.

The patterns of these 130 isolates are presented in Figure 3. The result of spoligotyping of the 130 isolates produced 49 distinct spoligotypes; 37 (30%) of them had a unique pattern. The remaining 93(71.5%) isolates were grouped into 12 clusters of strains possessing at least two isolates. The cluster size varied from 2 to 34 patients. Eighteen patterns of the 49 patterns were not previously reported. The most commonly occurring patterns were Spoligo International Type Number (SIT) 149, SIT53, and SIT37 each consisting of 34, 15, and 9 isolates, respectively, and these three strains accounted for 44.6% of the isolates. The most predominant lineage was Euro-American (lineage 4) consisting of 78.5% of the isolates while the lineages of 17.7% of the isolates were East African Indian (EAI) lineage (lineage 3).

## Discussion

In the present study, we evaluated the presence and magnitude of transmission of TB between Ethiopian farmers and their cattle. To achieve this study, TB-positive households and TB-negative households were included. In addition, cattle owned by both groups were investigated.

The result of this study indicated that neither transmission of *M*. *tuberculosis* from man to cattle nor of *M bovis* from cattle to man could be demonstrated. The main route of transmission of *M. bovis* is generally accepted to be through the milk which requires shedding of the organism by cow with tuberculous mastitis, which is a rare occurrence. Reports of *M*. *tuberculosis* among cattle exist [9]–[19] and cattle are likely to be exposed through inhalation of droplets of cough from active pulmonary TB cases of farmer and by ingestion of pasture contaminated with urine and sputum from infected farmer [11]–[14] but the exposure may not lead to disease. In general, it seems to be accepted that *M bovis* is substantially less virulent in humans than *M*. *tuberculosis* [28], conversely, *M*. *tuberculosis* is much less virulent in cattle than *M bovis* [29], although there is a need to confirm if transmission results in TB in cattle or only exposure and sensitization to tuberculin test. Our result showed larger number of skin test reactors in cattle owned by households with active TB.

In the present study, two *M. tuberculosis* strains of types SIT149 and SIT53 were isolated from five cattle. These two strains were the most commonly isolated strains from farmers who possess cattle. Together with isolates of SIT37, these made up 44.6% of the 130 isolates collected from patients keeping these cattle. This implies that these strains are present in the study area, and can be transmitted to cattle through different routes including ingestion of feed contaminated with infected sputum and/or urine from infected farmers. Similarly, the traditional animal husbandry practice of spitting tobacco juice into the oral cavity of cattle [19] could also be considered a means of transmission of *M. tuberculosis* from farmers to their cattle. The two *M. tuberculosis* cattle isolates were compared with the isolates from their respective owners. One of the owners (identification number 1180) could not provide sufficient sputum sample and was culture negative while the strain from his cattle was SIT53. The strain obtained from the second farmer (identification number 409) was different from the strain isolated from his cattle. The cattle strain was SIT149 while the strain isolated from the owner was new – octal number 777737377720771 and SIT number which matches to this strain was not found in the spoligotype database (spolDB4). Both two *M. tuberculosis* isolates were obtained from visible lesions with milder severity as compared to lesions caused by *M. bovis*. This implies that there could be non-visible lesions caused by *M. tuberculosis* which were not collected as they were not visible at post mortem examination. Of the 36 CIDT-positive cattle 11 had no visible lesions. These cattle might have been infected with *M. tuberculosis* or MTC. If we cultured lymph nodes with non-visible lesions, we might have isolated larger number of isolates of *M. tuberculosis* from cattle.

The most common spoligotype identified from farmers was the T family and the predominant lineage was the Euro-American. Similar to the present study, previous studies in Ethiopia showed that T and CAS genotypes were the dominant families [30], [31]. Nonetheless, no *M. bovis* was isolated from the TB positive farmers in this study. In agreement with result of the finding of the present study, recent studies in Ethiopia have reported transmission of *M. tuberculosis* from humans to cattle [18], [19].

From the interview, it was observed that the majority of study participant in both TB cases and TB free farmers consumed raw milk, and there was no association between consumption of raw milk and occurrence of human TB case. This was different from earlier reports, which associate raw milk consumption with extra pulmonary TB [32]–[34]. As indicated earlier, only 1% of the cows with TB excrete tubercle bacilli in their milk [35], which decreases probability of milk transmitting *M. bovis* to humans. In the present study, the milk was consumed at individual household, and thus has minimal role in transmitting the tubercle bacilli to people outside the farm in question. Inhalation could be an important route of transmission between farmers and cattle, further exacerbated by the low level of awareness of the farmers on the route of transmission and prevention of TB [36], [37]. The study participants were living in rural area and had poor housing condition, minimal access to health facilities, low awareness about disease, and usually shared house with their cattle. All these factors promote the transmission of TB between cattle and their owners. It was learnt that the local custom of spitting chewed tobacco or tobacco juice into the mouths of the cattle has animal husbandry significance. According to the respondents, animals fed on tobacco have good body condition and in a better health as compared to animals not fed on tobacco. As a result, this custom is widely been accepted and practiced by the community in the study area. Both men and women chew tobacco for this purpose.

The current study is not without limitations and the limitation of this study were (1) milk samples were not collected nor tested for the presence of mycobacterial pathogens; (2) the household survey did not collect data regarding consumption of soured milk vs. unpasteurized raw milk; (3) contamination of pasture with *M. tuberculosis* in sputum and tobacco juice (spit) was not demonstrated and (4) controls were required to be TB-negative at enrollment and have no history of TB+ household members for the previous 10 years. The 10 controls reporting “a TB+ family member” should not have been enrolled or should have been disqualified.

In conclusion, the present study did not identify any transmission of *M. tuberculosis* between humans and cattle. Similarly, no transmission of *M. bovis* between farmers and their cattle was found, even with 82% of households reporting consumption of unpasteurized milk produced by their animals. Herds and cattle belonging to 146 TB+ farmers had statistically significant increased rates of skin test reaction to the CIDT (14.4% vs. 4.3%; χ^2^ = 8.63, p<0,01) when compared to the 141 TB-negative control farmers and their herds, suggesting increased exposure and sensitization to mycobacteria. The practice of acidifying or “souring” milk prior to consumption can eliminate the risk of transmission if acidified to a pH <4.2, and may explain the absence of *M. bovis* infections in the TB+ farmers. When compared to controls, TB+ farmers were younger (34.7 years vs. 44.0 years; χ^2^ = 5.07, p<0.001), more likely to be female (49.3% vs. 16.3%; χ^2^ = 35.3; p<0,001), to share housing with their cattle (64% vs. 48%; χ^2^ = 7.93, p<0,01); and to have a TB+ family member (39.7% vs. 7.1%; χ^2^ = 41.9, p<0.001). Further study is needed to address the implications of these findings.
